# Prevalence and Diversity of Haemosporidian–Associated Matryoshka RNA Viruses in a Natural Population of Wild Birds

**DOI:** 10.1002/ece3.71239

**Published:** 2025-05-26

**Authors:** Carlos W. Esperanza, Caroline E. Faircloth, Scott W. Roy, Ravinder N. M. Sehgal

**Affiliations:** ^1^ College of Biological Science University of California Davis California USA; ^2^ Department of Biology San Francisco State University San Francisco California USA; ^3^ School of Biological Sciences The University of Edinburgh Edinburgh UK

**Keywords:** disease ecology, Haemosporidian parasites, parasite‐virus interactions, RNAseq, wildlife transcriptomics

## Abstract

Matryoshka RNA viruses (MaRNAV) have recently been identified in association with haemosporidian parasites infecting both humans and birds, suggesting a potential role in host–parasite interactions. However, their prevalence, diversity, and ecological significance in avian hosts remain poorly understood. To address this knowledge gap, we investigated MaRNAV in wild bird populations in the San Francisco Bay Area. To investigate this, we examined blood samples from wild birds and birds undergoing rehabilitation in the San Francisco Bay Area. Samples were screened for haemosporidian parasite infections followed by RNA sequencing (RNAseq) and reverse transcriptase (RT) PCR to detect MaRNAV. Our analyses identified two novel MaRNAVs (MaRNAV‐5 and ‐6) in various bird species harboring diverse *Haemoproteus* and *Leucocytozoon* species. MaRNAV‐5, associated with *Haemoproteus*, exhibited 71.3% amino acid identity to MaRNAV‐4 and was found across 15 passerine species. MaRNAV‐6, linked to *Leucocytozoon*, shared 72.9% identity with MaRNAV‐3 and was found in 4 raptor species. The prevalence was 44.79% for MaRNAV‐5 in infected passerines and 22.22% for MaRNAV‐6 in infected raptors. These viruses were not found in uninfected birds and were consistently only in birds infected with haemosporidian parasites. Sanger sequencing revealed a high similarity of viral sequences across different bird species. Our findings indicate a notable prevalence of MaRNAV among local wild birds, suggesting potential impacts on their health and ecology. We discuss several hypotheses for the transmission and ecological role of these viruses in the context of haemosporidian parasite–host interactions. Further research is needed to determine the impact of these viruses on avian systems.

## Graphical abstract

This study investigated the prevalence and diversity of Matryoshka RNA viruses (MaRNAV) in wild birds in the San Francisco Bay Area, California. Using RNA sequencing and RT‐PCR, we identified two novel MaRNAVs (MaRNAV‐5 and ‐6) associated with haemosporidian infections in passerines and raptors. These findings suggest a high prevalence of these viruses, indicating potential impacts on avian health and ecology.

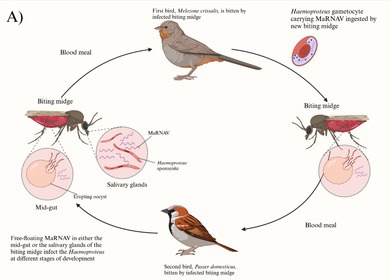

## 1 Introduction

Haemosporidian parasites (Order Apicomplexa; Genera: *Haemoproteus*, *Leucocytozoon*, *Plasmodium*) are a diverse group of single‐celled protozoa that infect a wide range of animal hosts (Garnham 1966; Telford Jr. 2009; Valkiūnas 2005). In vertebrate hosts, such as birds, these parasites invade red blood cells during one stage of their life cycle, leading to diseases such as avian malaria (Garnham 1966; Telford Jr. 2009; Valkiūnas 2005). However, their life cycle also involves other tissues and hosts, including insect vectors, which serve as definitive hosts for sexual reproduction and transmission (Valkiūnas 2005). These parasites have been extensively studied and found in most regions of the world (Valkiūnas 2005; Atkinson et al. 1995; Clark et al. 2014). Avian‐specific haemoporidian parasites encompass a diverse range of species within the genera *Plasmodium*, *Haemoproteus*, and *Leucocytozoon*. These genera are distinguished by their unique vectors, life cycles, and disease manifestations (Valkiūnas 2005). Despite these differences, acute parasitemia in an avian host may lead to avian malaria and similar malaria‐like infections, which have had some historically devastating impacts on wild bird populations, such as the extinction of several Hawaiian honeycreeper species, with no natural exposure or immunity to these diseases (Atkinson et al. 1995; Valkiūnas 2005). Typically, if individuals survive acute infection, they may live with less severe chronic infections for the rest of their lives, with varying amounts of subsequent parasite recurrence (Valkiūnas 2005; Himmel et al. 2024). However, the effects of chronic infections in avian hosts can be inconsistent, and prior infections may influence susceptibility to future haemospiridian parasite infections in different ways. Some lineages of haemosporidia show variation in infectivity, with haemosporidian parasites barely detectable in some bird species, while naïve bird communities may face a higher risk of severe infection (Palinauskas et al. 2008; Dimitrov et al. 2015). To better understand these variations in infectivity and their underlying mechanisms, recent genomic research on avian haemosporidia has made significant strides. This includes the complete genomic sequencing of Plasmodium relictum, as well as several analyses of the effects that parasitic infection has on hosts' transcriptomes through experimental inoculation (Ellis et al. 2022; Paxton et al. 2023; Videvall et al. 2020, 2021). While genomic research has advanced our understanding of haemosporidian parasites themselves, recent studies have also highlighted the potential role of viruses associated with these parasites in shaping host–parasite interactions. The discovery of viruses infecting parasitic protozoa has revealed that these viral associations can significantly influence parasite pathogenicity and host immune responses (Gómez‐Arreaza et al. 2017; Ives et al. 2011). Inspired by these findings, researchers have turned their attention to identifying and characterizing viruses associated with haemosporidian parasites. With the advancement of high‐throughput sequencing and bioinformatics, several novel viruses have been described in association with haemosporidian parasites by co‐occurrence (Charon et al. 2019; Rodrigues et al. 2022). However, despite these technological advances, little is known about the biology of these viruses or their ecological and evolutionary significance.

Of all the known viruses, RNA viruses make up most of all recognized viral species, with new viruses being described yearly (Forterre 2010; Woolhouse et al. 2013; Tsoleridis et al. 2019; Bejerman et al. 2020; Edgar et al. 2022; Petrone et al. 2024). Among the RNA viruses with the simplest genomes are the narnaviruses (Narnaviridae) (Dinan et al. 2020). Narnavirus genomes consist of a single‐segmented positive‐sense RNA (ssRNA), typically in the range of 2.3 to 3.6 kilobases (kb), that encodes an RNA‐dependent RNA polymerase (RdRp) (Dinan et al. 2020). While most narnavirus genomes are single‐segmented, recent findings have described more complex narnaviruses with additional putative segments, such as Culex narnavirus 1 (CxNV1) and Zhejiang mosquito virus 3, both being recovered from diverse mosquito hosts (Batson et al. 2021; Retallack et al. 2021). Some narnavirus genomes have the unique feature of being ambigrammatic, with the reverse complement of their genomic sequences coding large open reading frames (ORFs) (DeRisi et al. 2019; Retallack et al. 2021). Viruses that specifically infect parasitic protozoa are known as Parasitic Protozoan Viruses (PPVs) and are all single‐stranded (ss) or double‐stranded (ds) RNA viruses (Wang and Wang 1991). To date, PPVs have been characterized in *Trichomonas vaginalis*, *Giardia lamblia, Cryptosporidium parvum*, *Leishmania* spp., *Blechomonas* spp., and more recently, *Toxoplasmosis gondii* (Grybchuk et al. 2018; Gupta et al. 2024; Khramtsov et al. 1997; Wang and Wang 1986; Widmer et al. 1989). The presence of these PPVs can significantly alter aspects of their respective host parasite's pathogenicity by affecting the animal host's immune response (Gómez‐Arreaza et al. 2017; Heeren et al. 2023; Zhao et al. 2023; Gupta et al. 2024). Leishmania RNA virus 1 (LRV1), *Cryptosporidium parvum* virus 1 (CSpV1), and Trichomonas vaginalis virus (TVV), all in humans, have all been shown to weaken the host's defenses against their respective parasite by triggering a type I interferon (IFN) inflammatory response in their hosts (Ives et al. 2011; Fichorova et al. 2017; de Carvalho et al. 2019; Rada et al. 2022; Deng et al. 2023). On the other hand, some evidence shows that *Giardia lamblia* virus 1 (GlV1), also in humans, limits the growth of *G. lamblia* in its host, thereby mitigating infection (Miller et al. 1988). While these parasitic protozoan parasites have been detected in wild birds, there has been no evidence of their associated PPVs also being detected (McKenna 2010; Robinson et al. 2010; Shemshadi et al. 2015).

In 2019, the first PPVs associated with haemosporidian parasites were described (Charon et al. 2019). Taking a meta‐transcriptomic approach, blood samples collected from human patients in eastern Malaysia infected with various species of *Plasmodium*—*P. falciparum*, *P. vivax* , and *P. knowlesi* —and exhibiting clinical symptoms of malaria were tested (Charon et al. 2019). This led to the identification of a novel viral sequences encoding an RdRp and a hypothetical protein with no known function that were restricted to the *P. vivax* samples, suggesting that the virus was specific to *P. vivax* (Charon et al. 2019). Further analysis of additional meta‐transcriptomes from geographically diverse areas available on the NCBI SRA detected viral sequences that mapped to this RdRp and a second sequence, all restricted to *P. vivax* ‐infected samples. This novel virus was named Matryoshka RNA virus 1 (MaRNAV‐1) because of its Russian doll‐like nature of a virus infecting a parasite infecting a host, in this case an RNA virus infecting *P. vivax* infecting a human erythrocyte (Charon et al. 2019). Expanding their research to 12 *Leucocytozoon*‐infected Australian avian meta‐transcriptomes and using MaRNAV‐1 sequences as a reference, they described a second Matryoshka virus, MaRNAV‐2, detected in avian 8 samples infected with *Leucocytozoon* (Charon et al. 2019). In 2021, further metatranscriptomic research has since identified two additional MaRNAVs: MaRNAV‐3, associated with *Leucocytozoon*, and MaRNAV‐4, associated with *Haemoproteus* (Rodrigues et al. 2022). Phylogenetic analysis has revealed that the RdRps of these viruses are closely related to the RdRp of narnaviruses, with a major differentiating feature between the two viruses being a second RNA segment of unknown function found in MaRNAV‐1 and MaRNAV‐2 (Charon et al. 2019). Beyond the discovery of these viruses, virtually nothing is known about them, including their genomic organization, replication strategies, host range, and potential pathogenicity or ecological roles.

It can be construed that Matryoshka RNA viruses are specific to the haemosporidian parasite species with which they associate, given the evidence that MaRNAV‐1 was only detected in one species of *Plasmodium: P. vivax* (Charon et al. 2019). The discovery of MaRNAV‐2, ‐3, and ‐4 were performed without the aid of known haemosporidian parasite identification techniques, such as thin‐film microscopy (Ishtiaq et al. 2017). Because of this, the only information available regarding the hosts of these viruses is the definitive avian host species, parasite genus, and parasite lineages. MaRNAV‐3, a virus associated with *Leucocytozoon* parasites, was identified in a single bird transcriptome ( *Acanthis flammea* ), which was co‐infected with two cytochrome b lineages of the parasite genus *Leucocytozoon* (Galen et al. 2020; Rodrigues et al. 2022). MaRNAV‐4, associated with *Haemoproteus* parasites, was detected in two birds of the same species ( *Vireo plumbeus* ) found to be infected with *Haemoproteus* parasites of the lineages h‐VIRPLU01, h‐VIRPLU04, and h‐TROAE12 (Galen et al. 2020; Rodrigues et al. 2022). Due to the multiple lineage infections observed in these birds, determining the specific parasite‐virus association is not possible. However, since these viruses have only been detected in parasite‐infected samples, the findings suggest a potential specificity to the parasite genus or possibly to the insect vector. The prevalence of MaRNAV in wild avian populations, and their specificity to haemosporidian parasite infection, remains unknown. To fill this knowledge gap, we investigated MaRNAV prevalence and diversity using next‐generation sequencing and molecular techniques on blood samples collected from mist‐net‐captured birds in California, as well as from wild birds admitted to a local rehabilitation center. Birds are an excellent model for studying haemosporidian parasite ecology given their diverse host range, wide geographic distribution, and significant impact on populations, which can provide valuable insights into disease ecology and conservation (Valkiūnas 2005). Given that MaRNAV has been consistently detected in association with avian haemosporidian parasite infection, and the wide geographic distribution of previously detected MaRNAV, we hypothesize that (1) the prevalence of MaRNAV is positively associated with the prevalence of haemosporidian parasites, and (2) MaRNAV infection will be associated with the morphospecies of the haemosporidian parasite it infects. Moreover, given the vast diversity of haemosporidian parasites and their avian hosts, it is likely that additional MaRNAV lineages exist but have not yet been detected (Valkiūnas 2005). The geographic and ecological diversity of the San Francisco Bay Area, which includes a wide range of avian species and habitats, provides a unique opportunity to uncover novel viral diversity (Patten 1995). Understanding the role of MaRNAV in the avian‐haemosporidian parasite system can offer crucial knowledge for future studies on disease ecology and potential implications for bird conservation. For example, if MaRNAV can influence the pathogenicity or transmission dynamics of haemosporidian parasites, they could potentially exacerbate the impact of haemosporidian infections on bird populations. Identifying novel MaRNAV and understanding their interactions with parasites and hosts could inform conservation strategies by highlighting potential disease risks in vulnerable ecosystems. Additionally, this research can contribute to a broader understanding of how viruses interact with parasitic protozoans and influence their impact on both avian and human health.

## 2 Materials and Methods

### 2.1 Sample Collection

Field samples were collected from four regional parks and two urban parks around the San Francisco Bay Area, CA, over a period spanning October 7, 2022, to April 19, 2024, for a total of 32 days of sampling. Sampling occurred across multiple seasons, including fall (October–November 2022 and 2023), winter (December 2022, February–March 2023 and 2024), spring (March–April 2023 and 2024), and summer (June–August 2023). This extended timeframe allowed for the collection of data across varying environmental conditions and biological cycles. Each sampling day typically spanned from dawn to mid‐afternoon (approximately 6:00 AM to 3:00 PM), coinciding with peak bird activity. This schedule ensured consistent and effective sampling across all sites. The locations chosen for sampling were Lake Merced Park, San Francisco, (−122.486302, 37.7130597), Chain of Lakes Meadows, San Francisco, CA (−122.4983867, 37.7660642), Sunol Regional Wilderness, Sunol (−121.8817683, 37.5200063), Sibley Volcanic Regional Park, Oakland, (−122.2020921, 37.8596852), Tilden Regional Park, Orinda, (−122.2493329, 37.9006318), and Anthony Chabot Regional Park, Castro Valley (−122.0818417, 37.8596852) (Figure S1). 12‐m Japanese Nylon 36 mm mesh mist nets from Avinet Research Supplies (available at avinet.com) were used to capture birds in their natural environment, and 7–10 mist nets were placed at each location. Captured birds were aged and sexed based on morphology (plumage, coloration, bill measurements, tarsus) following Pyle (2008). If a bird could not be confidently aged or sexed using these criteria, the data were recorded as “unknown.” All birds were fitted with an aluminum alloy leg band provided by the US Fish and Wildlife Service. Banding data was submitted to the United States Geological Survey (USGS) Bird‐Banding Laboratory. All birds captured were checked for signs of extreme distress or exhaustion (open‐mouthed breathing, panting, wing‐drooping, or capture myopathy), signs of injuries (overt obvious injury such as a fractured bone or open wounds), or any abnormalities that would require their immediate release or need to enter wildlife rehabilitation.

A 25‐gauge needle was used to extract a blood sample (approximately 50 μL) from the brachial wing vein from each bird. Blood samples were used to make two thin blood smears, and the rest was distributed into two 1.5 mL cryogenic storage tubes, one containing 1 mL of Queen's Lysis Buffer for DNA preservation (Longmire et al. 1997), and one containing 500 μL of RNA later Stabilization Solution for RNA preservation. Samples were kept on dry ice until they could be stored in a ‐80°C freezer at the Avian Parasitology Laboratory at San Francisco State University (SFSU) for an average storage time of 2 months. Blood slides were air dried, immersed for around 30 s in absolute methanol for fixation, and stained using a 10% Giemsa solution according to the protocols described by Valkiūnas (2005). All slides were examined (two slides per bird) using a Nikon Eclipse 80i microscope and imaged using QCapture Pro v7.4.4.0 at 100× magnification. 100 fields were examined per slide.

To expand our search, additional blood samples were collected from larger birds, namely raptors admitted to the Lindsay Wildlife Experience (Walnut Creek, CA) for rehabilitation, hereafter referred to as museum samples. Sample collection lasted from November 4, 2022, until May 22, 2024, using the above‐mentioned methods to collect thin blood smears, as well as blood stored in Queen's Lysis Buffer. Samples were collected from the center every 1–2 months and kept at −20°C, and to account for shorter‐term storage at this temperature, blood was stored in 1 mL of Invitrogen TRIzol LS Reagent (Thermo Fisher Scientific, Waltham, MA), instead of RNA later. Each bird from this facility was only sampled once, and blood samples were picked up monthly from the center and transferred to the avian parasitology lab for −80°C storage.

### 2.2 Molecular Analysis

DNA was extracted from blood stored in Queen's Lysis Buffer using the Promega Wizard Genomic DNA Purification System. All isolated DNA from both field and museum samples was stored at −20°C, separately from the blood samples, until needed for analysis. Haemosporidian (*Plasmodium*, *Haemoproteus*, or *Leucocytozoon*) occurrence and species/lineage identification were detected using a combination of thin film light microscopy and PCR using the primers (HaemNFI/HaemNR3, HaemF/HaemR2, and HaemFL/HaemR2L; Table S2) and temperatures described by Bensch et al. (2000) and Hellgren et al. (2004). Amplified PCR products were sent to Elim BioPharm (Hayward, CA) for Sanger sequencing, and sequences were aligned using Geneious Prime 2024.0.4. Subsequent sequences were cross‐referenced to GenBank, as well as the MalAvi Avian Haemosporidian Database (Bensch et al. 2009).

### 2.3 RNA Seq & Transcriptome Assembly

Blood samples stored in RNA Later were frozen at −80°C until needed for RNA extraction. Before the extraction process, samples were incubated at room temperature for 15 min, centrifuged for 20 s at 12,000 × g, and the separated RNA Later was pipetted off. RNA extraction and isolation were performed using the Invitrogen PureLink RNA Mini Kit and treated with on‐column PureLink DNase Set. Samples stored in TRIzol were extracted using a phenol‐chloroform method, as per the Qiagen RNeasy Mini Kit and treated with Qiagen RNase‐Free DNase I. For all samples, a final elution of 30 μL was obtained and stored at −80°C. RNA quality and concentration were assessed using the Agilent 2100 Bioanalyzer at the SFSU Genomics, Transcriptomics, and Analysis Core (GTAC). Samples were chosen for sequencing if they met baseline requirements for RNA Integrity Numbers (RIN) of 5.5 or greater, and concentrations of 20 ng/uL or greater. RNA sequencing was performed by Novogene Co., LTD (Sacramento, CA) using the Illumina NovaSeq PE150 sequencing platform.

A total of 20 samples were selected for sequencing, including 8 *Leucocytozoon*‐infected samples, 2 *Haemoproteus*‐infected samples, 2 *Plasmodium*‐infected samples, and 8 uninfected (negative for both microscopy and PCR) samples. These samples were chosen at random but ensured that at least one sample representing each of the haemosporidian parasite genera infection (*Haemoproteus, Leucocytozoon*, *Plasmodium*) and a negative control group (uninfected) were included. Parasite composition and co‐infections were determined via a combination of PCR and microscopy Ishtiaq et al. 2017; however, it is still a possibility that co‐infection existed at a low enough volume that it was not detected by these methods. Paired‐end reads for each sample were generated in fastq format and released onto a remote server at SFSU. Trimmomatic v0.40 was used to trim adapter sequences from the raw reads, and the Trinity software v2.10.0 was used for *de novo* transcriptome assembly.

### 2.4 Viral Sequence Detection

A homology‐based approach was used to detect viral sequences in the assembled transcriptomes, as per Rodrigues et al. (2022). Diamond version 0.9.24 BLASTx was used for local sequence alignments against custom BLAST databases consisting of known MaRNAV RdRp protein sequences, as well as a database containing all the known RdRp protein sequences available on NCBI, using an overlap threshold of 30% identity and above, since previous MaRNAV searches found low percent identity between known and novel RdRps (Rodrigues et al. 2022). Hits were then submitted to NCBI BLASTx against the entire non‐redundant (nr) database to determine any similar sequences, as well as NCBI BLASTn to determine if the viral elements were integrated into the host's genomes. BLASTx hits were then submitted to the NCBI ORF finder. The longest ORFs were submitted to the Protein Homology/Analogy Recognition Engine version 2.0 (Phyre2) web portal, as well as HHPred Homology Detection Server (Zimmermann et al. 2018). HHpred is a tool for protein homology detection and structure prediction, while Phyre2 is used for predicting protein structure and function based on sequence alignment.

To characterize additional segments of these viruses, a separate Diamond BLASTx database was created that contained MaRNAV‐1 and MaRNAV‐2 hypothetical proteins discovered by Charon et al. (2019), and the same process as described above was repeated on all transcriptomes from this study, as well as the transcriptomes used by Rodrigues et al. (2022).

### 2.5 Complementary DNA (cDNA) and RT‐PCR

For Reverse Transcriptase PCR (RT‐PCR), Complementary DNA (cDNA) was made from all RNA extracts using the Invitrogen SuperScript IV Reverse Transcriptase (catalog #18090010), following the manufacturer's protocols. In short, 2 μL of RNA was combined with 1 μL of random hexamers (50 μM; catalog number: N8080127) and 9.8 μL of RNase‐free water in a 200 μL RNase‐free microcentrifuge tube. This solution was gently centrifuged for approximately 5 s, incubated at 65°C for 5 min in a thermocycler, and then on ice for at least 1 min. A mixture of the following was added to each sample while on ice: 4 μL of the SuperScript IV 5× Reaction Buffer, 1 μL of 10 mM dNTP, 1 μL of 0.1 M DTT, 1 μL of RNaseOUT (40 U/μL), and 1 μL of SuperScript IV Reverse Transcriptase (200 U/μL). This final mixture was gently mixed by pipetting the solution up and down several times and centrifuged for approximately 5 s. Samples were then incubated in a thermocycler at 23°C for 10 min, followed by 50°C for 1 h, and then 80°C for 10 min. Final cDNA samples were stored at −20°C until needed for PCR.

To validate the cDNA, oligo primers were created to amplify the phosphoglycerate kinase 1 (*PGK1*) gene, an avian reference gene as described by Olias et al. (2014) (Figure S2 and Table S2), and PCR was performed on all samples to amplify a 450 bp segment of this gene using primers pgk1_F (5′ CACCTTCCTCAAAGTGTCTCA 3′) and pgk1_R (5′ TGAAGTCAACAGGCAGAGTG 3′). The reaction mixture consisted of 25 μL total volume containing 2 μL cDNA template and 23 μL of PCR master mix. The thermocycler profile involved an initial denaturation at 94°C for 5 min, followed by 35 cycles of denaturation at 94°C for 30 s, annealing at 55°C for 30 s, extension at 72°C for 45 s, and a final extension at 72°C for 10 min.

MaRNAV primers were used as per Charon et al. (2019), and primers were developed for MaRNAV‐3, ‐4, and any novel MaRNAV sequences found via transcriptomics using the Primer 3 Plus Web Interface (Untergasser et al. 2007). All PCR reagents, amounts, concentrations, and temperature profiles are described in Data S1. All amplified products were Sanger sequenced through ELIM BioPharm, as described above, aligned in Genious Prime, and submitted to NCBI BLASTn.

### 2.6 Phylogenetics

Phylogenetics was used to further analyze novel MaRNAV RdRp sequences, and their relationship to all previously described MaRANV, as well as the 20 closest related narnavirus RdRp sequences. Protein sequences were retrieved from NCBI using accession numbers and made into a single fasta file, in addition to the MaRNAV‐5 and ‐6 sequences. The sequences were then aligned using MAFFT v7.309 E‐INS‐I algorithm, using the parameters –ep 0, –genafpair, and –maxiterate 1000 to align the sequences, formatted into Phylip|Phylip4 using BioPython, and input into IQ‐TREE with the bootstrap value set at 200 (−b 200) to assess the robustness of the tree (Nguyen et al. 2015). Nodes with high bootstrap values indicate strong support for the inferred relationship. A tree file in Newick format was obtained and loaded and edited on iTOL: Interactive Tree of Life (Letunic and Bork 2007).

### 2.7 Statistical Analysis

To investigate the relationship between haemosporidian parasite infection and MaRNAV‐5 infection, we used a generalized linear mixed model (GLMM) with a binomial distribution and logit link function, implemented using the lmer package (Kuznetsova et al. 2017). The Gaussian error distribution was confirmed using the DHARMa package (Hartig 2024). The dependent variable was MaRNAV‐5 infection status (binary: 0 = not infected, 1 = infected). The predictors included ‘any haemosporidian parasite infection’ (coded as a binary variable, 1 = any infection, 0 = no infection), parasite genus infection (*Haemoproteus*, *Leucocytozoon*, and *Plasmodium*, each coded as binary variables), as well as age (categorical) and weight (continuous, scaled). However, the variable ‘any_haemosporidian parasite infection’ perfectly predicted MaRNAV‐5, causing convergence issues with the GLMM, and was removed from the final model. To assess multicollinearity among predictors, we calculated the Variance Inflation Factor (VIF) using the car package in R (Fox and Weisberg 2019). All predictors had VIF values below 2, indicating no significant multicollinearity issues. We also evaluated whether the response variable (MaRNAV‐5 infection status) was zero‐inflated by comparing the observed distribution of zeros to the expected distribution under a binomial model using the *DHARMa* package. No evidence of zero‐inflation was detected, as the observed zeros were consistent with the binomial distribution. Model fit was evaluated using pseudo R‐squared (marginal) and a likelihood ratio test (LRT) comparing the full model to a null model (intercept only).

Initially, we considered including site as a random effect to account for potential variability across sampling locations. However, model fitting revealed that the variance of the random effect (site) was effectively zero (variance ≈ 6.7 × 10^−17^), indicating that site did not explain meaningful variability in the data. To address this, we simplified the model by removing the random effect and refitting it as a generalized linear model (GLM) with the same fixed effects. The GLM was fit using maximum likelihood estimation, and the significance of predictors was assessed using Wald z‐tests. All analyses were conducted in R (v4.4.1, R Core Team 2024) using the tidyverse and glm functions (Kuznetsova et al. 2017; Wickham et al. 2019).

Additionally, we performed chi‐squared tests to examine associations between virus presence and parasite infection. This analysis helped identify any significant relationships between the presence of MaRNAV and specific parasite infections.

To investigate the relationship between haemosporidian infection and MaRNAV‐6 infection, a similar generalized linear model (GLM) with a binomial distribution and logit link function was used. The dependent variable was MaRNAV‐6 infection status (binary: 0 = not infected, 1 = infected), and the predictors included parasite genus (*Haemoproteus*, *Leucocytozoon*, and *Plasmodium*, each coded as binary variables). To account for potential methodological variability due to different storage practices at one of the sites (museum vs. field‐captured), we performed the statistical analyses separately, using separate datasets for field and museum.

## 3 Results

### 3.1 Sample Collection & Parasite Prevalence

A total of 340 birds were caught using mist‐nets in the San Francisco Bay Area (Table S1). These birds belonged to various families, including Odontophoridae, Passerellidae, Parulidae, Regulidae, Corvidae, Tyrannidae, Turdidae, Hirundinidae, and Columbidae. Of these 340 field‐caught birds, 308 blood samples were collected; the remaining field‐caught birds were not sampled. While there were no birds that we caught that showed any major physical injury, some birds did get visibly stressed during handling and were subsequently not sampled. These birds were placed into a closed‐lid box for 10 min, re‐evaluated, and released. Additionally, 101 blood samples were collected from birds undergoing rehabilitation at the Lindsay Wildlife Museum, resulting in a total of 409 blood samples used for this study. This included birds belonging to the families Strigidae, Tytonidae, Accipitridae, Falconidae, Corvidae, and Cathartidae.

The overall prevalence of haemosporidian parasite infection in the field samples was determined to be 31.17% (*n* = 96) of all mist‐net‐caught samples, and 23.47% of the total (all birds tested: field‐caught and museum) blood samples tested (Table 1).

**TABLE 1:** Summary of haemosporidian prevalence.

Bird type	Infection type	Number of infected birds	Percentage of total samples
Field‐caught	*Haemoproteus*	59	14.23
Field‐caught	*Leucocytozoon*	43	10.51
Field‐caught	*Plasmodium*	19	4.65
Raptors	*Leucocytozoon*	62	15.16
Raptors	*Haemoproteus*	12	2.93

Among the field‐caught birds (*n* = 308), 59 were infected with *Haemoproteus* (19.16%), 43 were infected with *Leucocytozoon* (13.96%), and 19 were infected with *Plasmodium* (6.17%) (Table 1).

Of the museum samples (*n* = 101), 72 (71.29%) were infected with haemosporidian parasites, accounting for 17.60% of total blood samples. This included 62 museum samples infected with *Leucocytozoon* (61.39%) and 12 birds infected with *Haemoproteus* (11.88%). None of the museum samples were infected with *Plasmodium* (Table 1).

### 3.2 Novel MaRNAV Detection

Transcriptome sequencing yielded a total of 1,220,477,496 reads across all samples, with an average of 46,941,442 reads per sample (range: 25,536,100 to 71,926,410 reads). Using Diamond BLASTx, there were several hits (10 reads) detected in the transcriptome of an adult male California quail ( *Callipepla californica* ; accession SAMN43486645) from the field‐caught samples infected with *Haemoproteus lophortyx* (lineage h‐COLVIR03; Table 2). The top hit had a 71.4% amino acid identity to the RdRp of MaRNAV‐4 (E‐value = 0.0), and around 40%–47% identity to MaRNAV‐1, ‐2, and ‐3 RdRps. The hits varied in length but had high similarity to each other (95%–100%). The longest transcript sequence was submitted to the NCBI ORF finder, and the longest ORF (2925 nucleotides) was then submitted to Phyre2 and HHpred for a homology‐based search. Phyre2 reported the sequence had a 19% identity to an RNA‐dependent RNA polymerase with 91.4% confidence, and the HHpred search determined the sequence was an RNA‐directed RNA polymerase with 100% probability (E‐value = 2.8e‐51).

**TABLE 2:** Potential RdRp sequence homology summary.

MaRNAV	Avian host	Haemosporidian parasite genus and lineage	Diamond BLASTx hits and percent identity	Nucleotide length	Longest ORF	Phyre2 results	HHpred results
MaRNAV‐5	California Quail ( *Callipepla californica* )	*Haemoproteus lophortyx* (hCOLVIR03)	71.3% MaRNAV‐4 (DAZ89879.1), 46.2% Wilkie narna‐like Virus 1 (YP_009388589.1), 41.7% MaRNAV‐3 (DAZ89878.1), 40.4% MaRNAV‐1 (QGV56801.1), 44.6% MaRNAV‐2 (QGV56804.1)	2925 nt	974 aa	19% Identity to RNA‐dependent RNA polymerase (91.4% Confidence)	RNA‐directed RNA Polymerase (100% Probability, E‐value = 2.8e−51)
MaRNAV‐6	Barn Owl ( *Tyto alba* )	*Leucocytozoon californicus* (lBNOW04)	72.9% MaRNAV‐3 (DAZ89878.1), 62.7% MaRNAV‐2 (QGV56804.1), 60.4% MaRNAV‐1 (QGV56800.1), 44.4% MaRNAV‐4 (DAZ89879.1), 43.7% Wilkie narna‐like Virus 1 (YP_009388589.1)	2112 nt	703 aa	33% Identity to RNA‐dependent RNA polymerase (88.4% Confidence)	RNA‐directed RNA polymerase (100% Probability, E‐value = 2.2e−31)

The second positive results (> 100 reads) were detected in 3 Barn owls ( *Tyto alba* ; accessions SAMN43486652, SAMN43486653, SAMN43486655) transcriptomes from the museum samples, and were infected with *Leucocytozoon californicus* (l‐BNOW04; Table 2) (Walther et al. 2016). The top Diamond BLASTx hits shared a 72.9% amino acid identity to MaRNAV‐3 RdRp, also detected in a *Leucocytozoon*‐infected transcriptome, and 43%–63% identity to MaRNAV‐1, ‐2, and ‐4. Similarly, this sequence was submitted to the NCBI ORF finder, Phyre2, and HHpred, and the longest ORF (2112 nucleotides) was determined to be 33% identical to known RNA‐dependent RNA‐polymerases (phyre2: 88.4% confidence, HHpred: 100% probability, E‐value = 2.2e−31). This sequence was named Matryoshka RNA Virus 6 (MaRNAV‐6).

Both MaRNAV‐5 and ‐6 were added to the initial reference BLASTx database, and the transcriptomes were re‐searched. The MaRNAV‐6 RdRp sequence was detected in two other Barn owls, with ≥ 95% nucleotide identity to the reference sequence, which were also infected with *L. californicus* . MaRNAV‐1, ‐2, ‐3, ‐4, and ‐5 were not detected in any of the other transcriptomes.

Hypothetical protein sequences were not found in any transcriptome where MaRNAV‐4 or MaRNAV‐5 were found. The MaRNAV‐3 hypothetical protein is 298 amino‐acids long and has a 29.8% pairwise identity to MaRNAV‐2 hypothetical protein (QGV56802.1). MaRNAV‐6 hypothetical protein was found to be 302 amino‐acids long and has a 34.1% identity to MaRNAV‐2 hypothetical protein.

### 3.3 Matryoshka RNA Virus Prevalence

Forty‐three of the samples collected from the field were found to be infected with MaRNAV‐5, accounting for 44.79% of field samples infected with haemosporidian parasites, 13.96% of all field samples, and 10.51% of all samples used in this study (Table 3). All 43 samples were field‐caught birds belonging to different families and were infected with *Haemoproteus* parasites, with 13 harboring a co‐infection of *Leucocytozoon* and 1 co‐infection with *Plasmodium*. The virus was detected across 15 different bird species, harboring different *Haemoproteus* lineages that are specific to their intermediate avian host (Table 3). Because of the outliers of field‐caught birds being co‐infected with multiple haemosporidian parasites, we could not make a strong association between *Haemoproteus* and MaRNAV‐5, despite MaRNAV‐5 only being found in all *Haemoproteus*‐infected field‐caught birds (χ^2^ *p* = 0.147).

**TABLE 3:** Summary of bird species harboring haemosporidian parasites, their lineages, and MaRNAV‐5 or MaRNAV‐6.

Bird species (*n*)	Parasite genus	Parasite lineages	MaRNAV‐5	MaRNAV‐6
*Junco hyemalis* (110)	*Haemoproteus*	hGYMSAL01	4	0
		hJUHYE03	4	0
	*Haemoproteus + Leucocytozoon*	hGYMSAL01, lSTOCC16	3	0
		hGYMSAL01, lDENCORE05	1	0
		hGYMSAL01, lCNEORN01	1	0
	*Haemoproteus + Plasmodium*	hGYMSAL01, pMOLATE01	1	0
*Setophaga coronata* (17)	*Haemoproteus + Leucocytozoon*	hGYMSAL01, lCB1	3	0
*Poecile rufescens* (28)	*Haemoproteus*	hPASILI01	2	0
	*Haemoproteus + Leucocytozoon*	hPASILI01, lROF6	2	0
*Callipepla californica* (3)	*Haemoproteus*	hCOLVIR03	2	0
*Melospiza melodia* (18)	*Haemoproteus*	hDENCORE03	4	0
*Melozone crissalis* (6)	*Haemoproteus*	hTABI02	1	0
	*Haemoproteus + Leucocytozoon*	hROFI1, lDENCORE05	1	0
		hJUHYE03, lZOLEU02	1	0
*Pipilo maculatus* (7)	*Haemoproteus*	hCATUST10	1	0
		hJUHYE03	1	0
*Catharus guttatus* (17)	*Haemoproteus*	hCATUST22	1	0
*Setophaga townsendi* (2)	*Haemoproteus*	hTABI02	1	0
*Zonotrichia atricapilla* (28)	*Haemoproteus*	hDUNNO01	1	0
*Baeolophus inornatus* (2)	*Haemoproteus*	hVIGIL08	1	0
*Haemorhous purpureus* (9)	*Haemoproteus*	hGYMSAL01	2	0
*Turdus migratorius* (2)	*Haemoproteus + Leucocytozoon*	hCATUST22, lTUMIG11	1	0
*Troglodytes aedon* (1)	*Haemoproteus + Leucocytozoon*	hMAFUS02, lJUHYE16	1	0
*Haemorhous mexicanus* (8)	*Haemoproteus*	hSISKIN1	1	0
	*Haemoproteus + Leucocytozoon*	hPIPMAC01, lCB1	1	0
*Tyto alba* (28)	*Leucocytozoon*	lBNOW04	0	10
*Bubo virginianus* (33)	*Leucocytozoon*	lSTOCC16	0	2
*Elanus leucurus* (3)	*Leucocytozoon*	lBNOW04	0	2
*Accipiter cooperii* (1)	*Leucocytozoon*	lBNOW04	0	1
*Buteo lineatus* (5)	*Leucocytozoon*	lSTOCC16	0	1

Sixteen museum samples were found to be infected with MaRNAV‐6, accounting for 22.22% of infected museum samples, 15.84% of all museum samples, and 3.91% of total samples (Table 3). All samples were from raptors infected with *Leucocytozoon*, including two that were co‐infected with *Haemoproteus*. Similar to MaRNAV‐5, MaRNAV‐6 was detected across various museum bird species harboring different *Leucocytozoon* infections. None of the museum samples tested positive for MaRNAV‐5, and none of the field‐caught samples tested positive for MaRNAV‐6. No sample, field‐caught or museum, tested positive for MaRNAV‐1, ‐2, ‐3, or ‐4. Further, MaRNAV presence was solely detected in haemosporidian‐infected bird samples, and no uninfected samples (*n* = 241) ever tested positive for MaRNAV infection, indicating a strong association between MaRNAV and haemosporidia infection (χ^2^ *p* = 0). Each sample was tested at least 3 times to test for false positives using cDNA technical replicates made from the same RNA isolates. Only samples that were continuously tested positive for MaRNAV (two or more RT‐PCRs yielded positive results) were considered true positives.

### 3.4 Statistical Analysis

We fit a generalized linear model (GLM) with a binomial distribution and logit link function to assess the relationship between haemosporidian infection and MaRNAV‐5 and ‐6 infection. For the field samples, *Haemoproteus* infection was significantly associated with higher odds of MaRNAV‐5 infection (β = 5.54, SE = 0.84, z = 6.58, *p* < 0.001; Table 4). *Leucocytozoon* infection also showed a significant positive association with MaRNAV‐5 infection (β = 1.57, SE = 0.66, z = 2.38, *p* = 0.017; Table 4). In contrast, *Plasmodium* infection (β = 0.83, SE = 1.51, z = 0.55, *p* = 0.584), age (*p* > 0.05 for all categories), and weight (β = 0.11, SE = 0.16, z = 0.67, *p* = 0.506) were not significant predictors of MaRNAV‐5 infection. The model explained a substantial proportion of the deviance in MaRNAV‐5 infection, with a residual deviance of 100.42 on 309° of freedom, compared to a null deviance of 251.39 on 315° of freedom. A likelihood ratio test comparing the full model to a null model (intercept only) indicated that the predictors significantly improved model fit (χ^2^ = 150.97, df = 6, *p* < 0.001; Table 4). The marginal R‐squared value, calculated using the delta method, was 0.410, indicating that approximately 41.0% of the variance in MaRNAV‐5 infection was explained by the predictors in the model.

**TABLE 4:** Results of generalized linear models (GLMs) predicting MaRNAV infection in two datasets.

Dataset	Term	Estimate	Standard error	*z*‐value	*p*‐value	95% CI (odds ratio)
Field	(Intercept)	−5.484	0.85	−6.48	< 0.001***	(0.001, 0.027)
Field	*Haemoproteus*	5.537	0.84	6.58	< 0.001***	(48.876, 1316.123)
Field	*Leucocytozoon*	1.566	0.66	2.38	0.017*	(1.319, 17.385)
Field	*Plasmodium*	0.826	1.51	0.55	0.584	(0.118, 44.123)
Field	Age (Other)	1.615	1.18	1.37	0.17	(0.503, 50.234)
Field	Age (unknown)	0.617	1.10	0.56	0.576	(0.214, 16.023)
Field	Weight	0.107	0.16	0.66	0.506	(0.813, 1.523)
Museum	(Intercept)	−4.126	1.44	−2.87	0.004**	(0.001, 0.267)
Museum	*Haemoproteus*	0.037	0.81	0.05	0.963	(0.213, 5.045)
Museum	*Leucocytozoon*	2.868	1.47	1.96	0.050*	(1.002, 308.262)

Model fit statistics (combined)		
Dataset	Statistic	Value
Field	Null deviance	251.39 (df = 315)
Field	Residual deviance	100.42 (df = 309)
Field	AIC	114.42
Field	Marginal *R* ^2˛^ (delta)	0.41
Field	LRT statistic	150.97 (df = 6)
Field	LRT *p*‐value	< 0.001***
Museum	Null deviance	84.87 (df = 100)
Museum	Residual deviance	73.77 (df = 98)
Museum	AIC	79.77
Museum	Marginal *R* ^2˛^ (delta)	0.896
Museum	LRT statistic	11.10 (df = 2)
Museum	LRT *p*‐value	0.004**

*Note:* This table summarizes the results of generalized linear models (GLMs) with a binomial distribution and logit link function, used to assess the relationship between haemosporidian infection and MaRNAV infection in two datasets: Field and museum. For each dataset, the table provides the estimated coefficients, standard errors, *z*‐values, *p*‐values, and 95% confidence intervals for the predictors. Model fit statistics, including null deviance, residual deviance, AIC, marginal *R* ^2^ (delta), likelihood ratio test (LRT) statistic, and LRT *p*‐value, are also reported.

* *p* < 0.05.

** *p* < 0.01.

*** *p* < 0.001.

The museum model results indicated that *Leucocytozoon* was a marginally significant predictor of MaRNAV‐6 infection (β = 2.87, SE = 1.46, z = 1.96, *p* = 0.050), while *Haemoproteus* was not significant (β = 0.04, SE = 0.81, z = 0.05, *p* = 0.963; Table 4). The intercept term was significant (β = −4.13, SE = 1.44, z = −2.87, *p* = 0.004), indicating that the baseline probability of MaRNAV‐6 infection was significantly different from zero. The model explained a substantial proportion of the deviance in MaRNAV‐6 infection, with a residual deviance of 73.77 on 98° of freedom, compared to a null deviance of 84.87 on 100° of freedom. A likelihood ratio test indicated that the predictors significantly improved model fit (χ^2^ = 11.10, df = 2, *p* = 0.004). The marginal R‐squared value, calculated using the delta method, was 0.896, indicating that approximately 89.6% of the variance in MaRNAV‐6 infection was explained by the predictors in the model. These results align with the findings from the chi‐squared test, which revealed a significant association between *Leucocytozoon* and MaRNAV‐6 infection (χ^2^ = X, df = X, *p* < 0.001).

### 3.5 Sanger Sequence Analysis

All amplified Sanger sequences showed high nucleotide alignment similarity to MaRNAV‐5 and MaRNAV‐6 RdRp sequences (*p* < 0.05), despite being detected across different bird and parasite species (Figure 2). MaRNAV‐5 was found in birds of different orders, and this assemblage of bird samples also harbored very distinct lineages of *Haemoproteus* (Table 4). NCBI BLASTx and BLASTn results consistently matched the sequences of MaRNAV‐6 and MaRNAV‐5 to MaRNAV‐3 and MaRNAV‐4, respectively.

## 4 Discussion

The discovery of haemosporidian parasite‐associated viruses raises crucial questions about their potential impact on parasite virulence and host health. Here, we aimed to investigate the prevalence and diversity of novel Matryoshka RNA viruses (MaRNAV) in a wild bird community and determine their association with avian haemosporidian parasite infection. To our knowledge, this study is the first of its kind to use living bird populations to detect MaRNAV and provides valuable insights into the prevalence and diversity of MaRNAV in a local avian community. Using molecular methods and transcriptomics, we identified two novel MaRNAV: MaRNAV‐5, detected in birds infected with *Haemoproteus* parasites, and MaRNAV‐6, associated with *Leucocytozoon* parasites. The results from the present study show similarities to the only other existing investigation into novel RNA viruses (Rodrigues et al. 2022). These findings reveal a strong correlation between haemosporidian parasite infection and viral infection because MaRNAV were exclusively detected in haemosporidian‐infected birds, suggesting a close association between the two. These viruses are moderately prevalent within the haemosporidian‐infected bird communities around the San Francisco Bay Area, with between 22.22% and 44.79% of haemosporidian parasite‐infected birds harboring these viruses, supporting our hypothesis of viral prevalence correlating with parasite infection.

**FIGURE 1:**
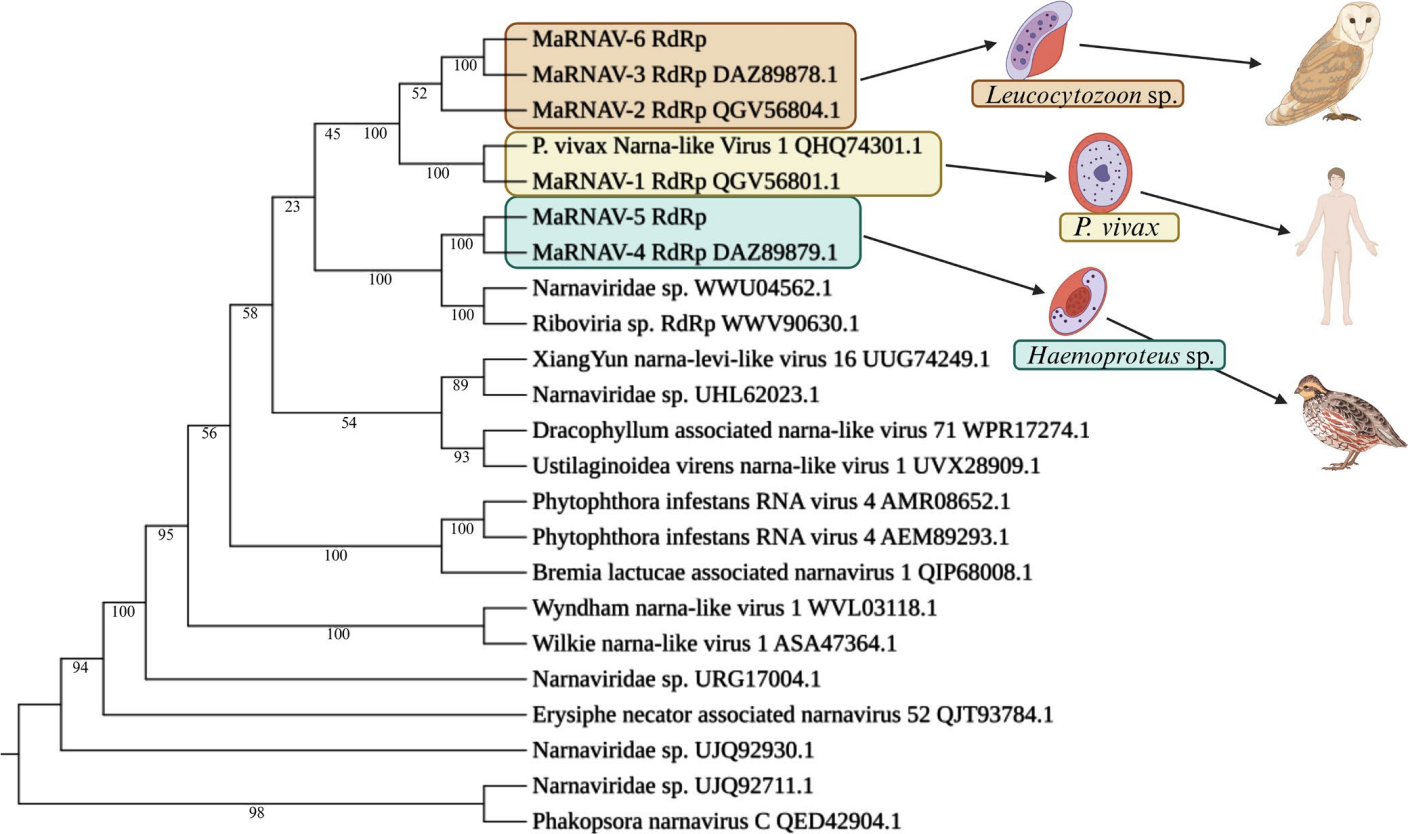
Phylogenetic tree showing how the protein sequences were aligned using the E‐INS‐I algorithm in the MAFFT multiple sequence alignment program (v7.309) and put into IQ‐tree (v1.6.10) with 200 bootstraps (B = 200). IQ‐tree chose the LG + F + I + G4 model according to BIC. Edited using BioRender.

Phylogenetic analysis of the MaRNAV RdRp region, as well as closely related RdRps, shows that MaRNAV‐1, ‐2, ‐3, and ‐6 cluster together, forming their own distinct clade (Figure 1). Unsurprisingly, the *Leucocytozoon*‐associated MaRNAVs clustered together. The divergent nature of these viruses further prompts a potential reclassification of these viruses as a new genus or viral family. However, MaRNAV‐4 and ‐5 clustered with narnavirus and ribovirus previously detected in bat metagenomes (WWU04562.1, WWV90630.1). This suggests that the *Haemoproteus*‐associated MaRNAV may be more closely related to the canonical narnaviruses, even potentially being narnaviruses themselves. Further evidence of this was our inability to detect the putative second protein segments of MaRNAV‐4 or MaRNAV‐5, a key characteristic of MaRNAV that distinguishes them from narnaviruses, leaving them to be more characteristic of the single‐segmented narnaviruses.

**FIGURE 2:**
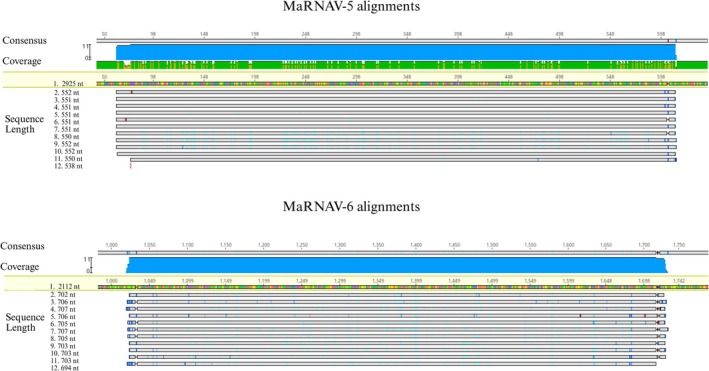
Sanger sequence alignment of amplified PCR products with MaRNAV‐5 (top) and MaRNAV‐6 (bottom) RdRp sequences. The beige colored lines, labeled as number 1 in both top and bottom, represent the section of the respective RdRp's amplified by PCR. Each gray line below the RdRp sequence represents a trimmed and aligned sequence that was amplified via RT‐PCR. The numbers on the left column represent the nucleotide length for each sequence. The target for MaRNAV‐5 primers was a 550 bp sequence, and MaRNAV‐6 targeted a 700 bp sequence. Each sequence was trimmed and edited for quality, and alignment was performed using Geneious Prime 2024.0.4.

**FIGURE 3:**
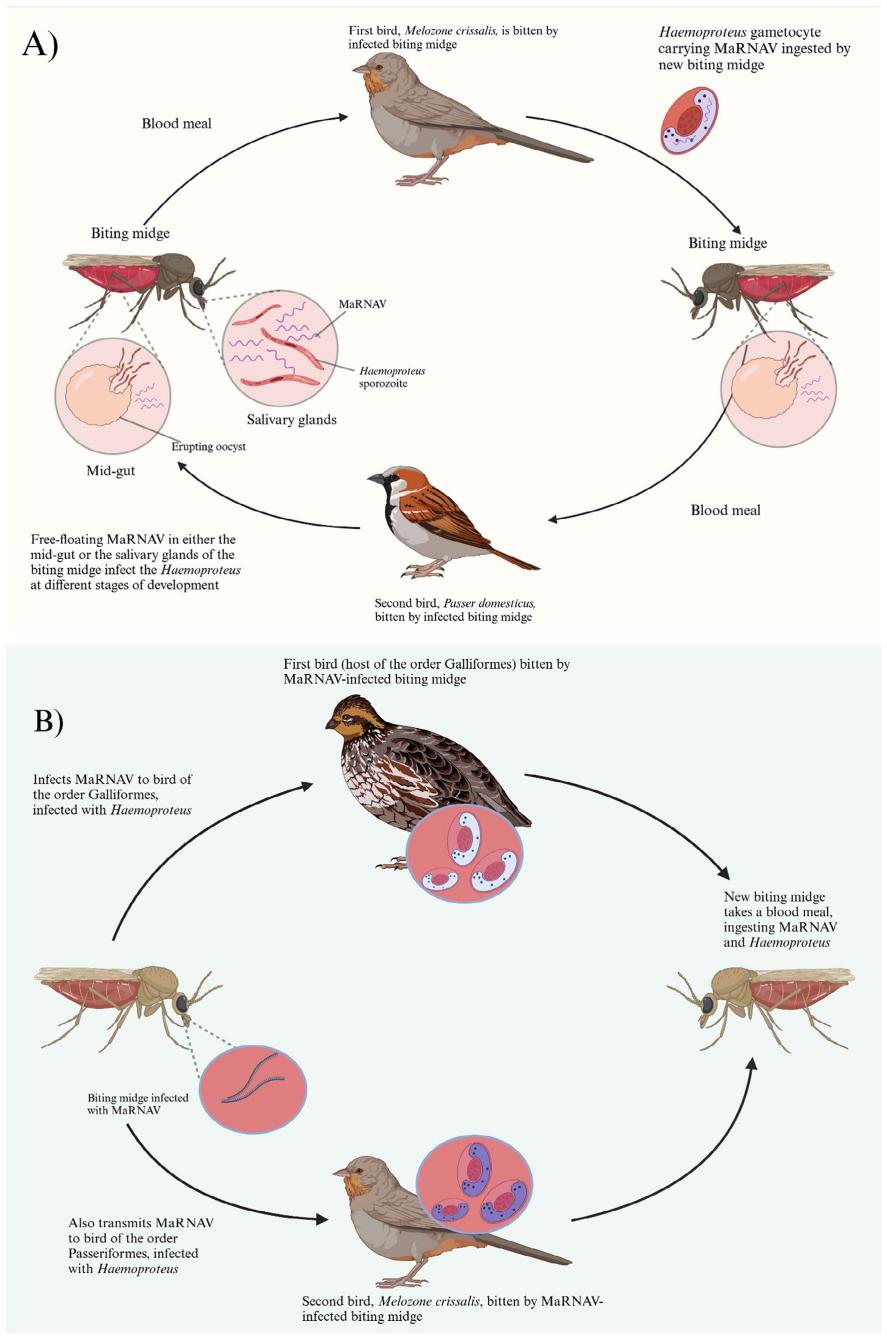
Hypothetical transmission cycle for Matryoshka RNA viruses, using MaRNAV‐5, *Haemoproteus*, and biting midges as an example. (A) Depicts the insect vector as the definitive host as the virus and utilizes transmission of the haemosporidian parasites as a “vector” for transmission to the next insect vector. The first bird, *Melozone crissalis* , is bitten by a biting midge infected with *Haemoproteus* and MaRNAV, becoming infected with both. The next biting midge to bite the first bird ingests the *Haemoproteus* carrying the MaRNAV, ready to transmit and infect the second bird, *Passer domesticus* . (B) Depicts MaRNAV transmission independent of haemosporidian to various avian species infected with various haemosporidian species. In this scenario, a biting midge infected with MaRNAV takes a blood meal from two birds belonging to different orders: Galliformes and Passeriformes, both infected with *Haemoproteus*. The now coinfected birds are then bitten by an uninfected biting midge, which ingests both *Haemoproteus* and MaRNAV.

### 4.1 Viral Transmission

An important question pertinent to the biology of the MaRNAV regards how they are transmitted. Previous research suggested that the most likely scenarios involve co‐transmission of the virus and the haemosporidian parasite through vertical transmission (Charon et al. 2019). In the current study, the detection of novel MaRNAV in multiple avian species, each harboring different parasite lineages, challenges this previous hypothesis (Charon et al. 2019). These results suggest that interspecies horizontal viral transmission could play a significant role in the spread of MaRNAV (Rubbenstroth et al. 2016). Several potential explanations could account for the patterns observed in this study. The most straightforward explanation is vector‐mediated horizontal transmission. The vectors of haemosporidian parasites—such as mosquitoes, biting midges, and black flies (Valkiūnas 2005)—may also independently carry and transmit MaRNAV to different bird species. Black flies and biting midges have been shown to be vectors of Vesicular Stomatitis Virus and Arborvirus, respectively (Mead et al. 1999; Mellor et al. 2000; Drolet et al. 2021; Sick et al. 2019; Kampen and Werner 2023). Additionally, mosquitoes are well‐known vectors of arboviruses that cause such diseases as dengue fever, chikungunya, and Zika (Soni et al. 2023).

Here, we posit the hypothesis that the insect is the focal host for the virus, and the virus may use the haemosporidian parasite as a vector to facilitate its transmission to the next insect host (Figure 3A). The reason for this hypothesis is that nearly identical viral RdRp sequences were found in a wide array of bird species, even from different orders. For example, MaRNAV‐5 was first found in a California quail ( *C. californica* ) of the order Galliformes. RT‐PCR then detected the RdRp of MaRNAV‐5 in a Dark‐eyed junco (*Junco*. *hyemalis*) and a California towhee ( *Melozone crissalis* ), both of the order Passeriformes. These different bird species and orders carry a broad number of haemosporidian parasite lineages (Figure S3). However, it is known that both biting midges and blackflies routinely feed on many bird species (Valkiūnas 2005). Thus, it appears that the virus has developed more specificity towards the insect hosts, which are indeed the definitive hosts of the haemosporidian parasites (Valkiūnas 2005). A potential explanation for why MaRNAV‐6 was only associated with *Leucocytozoon* in raptors may be linked to blackfly host‐specificity, as described by Malmqvist et al. (2004). In this study, 200 engorged blackflies from 17 different species were analyzed, and several distinct patterns of host preference emerged. First, they found that there was a clear separation of blackflies that prefer mammalian blood meals and avian blood meals. Within each, mammalian and avian, they found even more specificity, with specific species of blackflies feeding on specific host types. For example, *Simulium annulus* fed on cranes, *Simulium dogieli* on ducks, and *Simulium silvestre* fed on thrushes. The second pattern that they found was that blackflies preferred to feed on larger hosts.

However, it is also possible that the observed pattern reflects a case where one infection increases susceptibility to another, and that the virus infection might act as more opportunistic. Individuals solely infected with the virus could potentially fight off the infection more effectively, but when combined with haemosporidian parasites, their ability to do so might be significantly compromised. This interaction could make it more challenging for the host to manage the co‐infection. This alternative hypothesis suggests that the presence of haemosporidian parasites may enhance the susceptibility of the insect hosts to viral infection. Given the speculative nature of these hypotheses and the lack of experimental data to determine the sequence of infections, further studies are needed to explore these possibilities. A thorough investigation of both the midgut and salivary glands of the invertebrate will also be necessary to validate this hypothesis.

As an alternative hypothesis, the biting midges that carry and transmit *Haemoproteus* may independently carry MaRNAV‐5 and transmit the virus to various birds already infected with different species of *Haemoproteus* (Figure 3B). Likewise, this applies to blackflies that carry *Leucocytozoon*. The ecological overlap between the vectors and avian hosts (shared habitats, seasonal activity) may facilitate this hypothetical co‐infection process. MaRNAV‐5 was detected in birds of different orders (Galliformes, Passeriformes) across distinct geographic locations, all of which were infected with *Haemoproteus* parasites but harbored unique parasite lineages (Figure S3). Specifically, MaRNAV‐5 was first detected in a California quail (order Galliformes) infected with *Haemoproteus* lineage hCOLVIR03, captured in Anthony Chabot Park (Castro Valley, CA). The same virus was also detected in a Dark‐eyed junco (order Passeriformes) infected with *Haemoproteus* lineage hJUHYE03, captured in Tilden Regional Park (Orinda, CA), and in a Song sparrow (order Passeriformes) infected with *Haemoproteus* lineage hDENCORNA3, captured in Golden Gate Park (San Francisco, CA). This suggests that MaRNAV‐5 can infect birds across different genera and orders, despite the variability in Haemosporidian parasite lineages and geographic locations.

In contrast, MaRNAV‐6 was detected exclusively in birds infected with *Leucocytozoon* and was restricted to birds outside the San Francisco Bay Area. For instance, MaRNAV‐6 was found in Barn owls, Great horned owls, Red‐tailed hawks, and Red‐shouldered hawks that were brought to a wildlife hospital in Walnut Creek after being found in cities such as Livermore, Dublin, Fairfield, Vacaville, and Antioch. None of the MaRNAV‐6‐positive birds were captured within the immediate San Francisco Bay Area, further underscoring the geographic variability and potential separation in MaRNAV prevalence. This geographic separation suggests that ecological factors, such as habitat type and vector distribution, may influence the transmission dynamics of MaRNAV‐5 and MaRNAV‐6. The results from this study, however, point to the first scenario as a much more likely hypothesis, as all MaRNAV were found solely in haemosporidian‐infected samples, so if an insect vector infected with MaRNAV were transmitting the virus independent of haemosporidian infection, avian samples uninfected with haemosporidian parasites should have also tested positive for MaRNAV.

It is possible, and likely, that this analysis missed other novel MaRNAV, and that the prevalence and diversity of these viruses are much higher than detected in this study. This study was only able to detect novel MaRNAV through meta‐transcriptomic analysis, despite searching for previously detected MaRNAV. Given the relatively small sample size (*n* = 20), it is likely that more sequencing would have led to the discovery of more MaRNAV, potentially in hosts uninfected with haemosporidian parasites. This scenario, however, would be unlikely given the results from the current study, as well as previous MaRNAV investigations (Charon et al. 2019; Rodrigues et al. 2022).

The effect that these viruses have on their host remains a major unknown. Considering how prevalent they are in nature, it will be essential to study how viral presence affects parasite pathogenicity, if at all. The rapid mutation of RNA viruses can significantly change their functionality, including their virulence, or the parasite's pathogenicity (Steinhauer and Holland 1987; Moya et al. 2000; Furio et al. 2005; Duffy et al. 2008). There are three potential ways in which MaRNAV might influence haemosporidian parasite infection in the host. First, the virus could increase pathogenicity by triggering a type I IFN response, as observed with LRV1, CSpV1, and TVV (Ives et al. 2011; Fichorova et al. 2017; de Carvalho et al. 2019; Rada et al. 2022; Deng et al. 2023). Second, MaRNAV might reduce parasite pathogenicity, similar to the effects of GIV1 (Miller et al. 1988). Third, MaRNAV may have no impact on parasite pathogenicity in the host at all. These hypotheses could be tested through experimental inoculation of birds with MaRNAV and haemosporidian parasite‐infected blood, followed by analysis of gene expression differences among these birds, healthy birds, and those infected with haemosporidian parasites but not MaRNAV.

It is still unknown whether the viruses infect the haemosporidian parasite cell or the animal host cell. All previous data suggest that the virus is at least associated with haemosporidia, but it has yet to be proven to be infecting the haemosporidian parasite. Uncovering this mystery could potentially give some insight into the transmission and life cycle of the MaRNAV. For example, finding that the MaRNAV does not infect the haemosporidian parasite cells directly increases the possibility of horizontal transmission rather than vertical. Future studies could implement RNA Scope In Situ Hybridization (ISH) to locate and visualize the RdRp within the cell. Usage of this technology in the realm of avian haemosporidian parasites opens the possibilities of realistically localizing MaRNAV infections.

The discovery of MaRNAV, and their relationship to haemosporidian parasites could have significant implications for the future of malaria and avian malaria research. If MaRNAV can affect haemosprodian parasite pathogenicity, new disease control and prevention strategies may need to be introduced. For example, understanding the mechanism in which MaRNAV alters haemosporidian parasite virulence, or host response, can lead to novel therapeutics that target the virus, or the parasite *and* the virus. It is crucial for future work to investigate the insect vector species for the presence of the virus and identify if the virus is more broadly disseminated in the insect's body. By understanding these complex interactions between MaRNAV, haemosporidian parasites, and their insect vectors, we can gain invaluable insights that may inform future strategies for disease control, prevention, and wildlife conservation.

## Author Contributions

**Caroline E. Faircloth:** data curation (equal), formal analysis (equal), investigation (equal), writing – review and editing (supporting). **Carlos W. Esperanza:** conceptualization (equal), data curation (lead), formal analysis (lead), investigation (equal), methodology (lead), visualization (lead), writing – original draft (lead), writing – review and editing (lead). **Scott W. Roy:** formal analysis (supporting), methodology (supporting), supervision (equal), writing – review and editing (supporting). **Ravinder N. M. Sehgal:** conceptualization (equal), formal analysis (equal), funding acquisition (lead), methodology (lead), supervision (lead), validation (supporting), writing – original draft (supporting), writing – review and editing (supporting).

## Ethics Statement

All field bird handling and sampling protocols and procedures were conducted in accordance with federal, state, and institutional guidelines for the ethical treatment of wildlife. The research was authorized under the United States Department of the Interior, US Geological Survey Permit No. 23555, which allowed for the federal leg banding, taking, possession, and transportation of blood and feather samples, the use of audio lures, baited traps, mist nets, walk‐in traps, and other trapping methods. Additional authorization was granted by the State of California Natural Resources Agency Department of Fish and Wildlife under Federal Permit No. S‐200730003‐20,073‐001‐01. All procedures were reviewed and approved by the Institutional Animal Care and Use Committee (IACUC) at San Francisco State University under Protocol No. A2024‐16, titled “Molecular Genetics of Avian Malaria Viruses.” These permits ensured compliance with all relevant ethical standards for the care and use of animals in research, and every effort was made to minimize distress and discomfort to the birds involved in this study.

## Conflicts of Interest

The authors declare no conflicts of interest.

## Supporting information


Figure S1.

Figure S2.

Figure S3.

Data S1.

Table S1.

Table S2.

## Data Availability

Raw RNA sequencing data was submitted to the NCBI SRA under BioProject PRJNA1156234. MaRNAV‐5 and ‐6 sequences have been submitted to GenBank, accession numbers: XJW06183.1, XJW06184.1, XJW06185.1, XJW06186.1.
