# Correction to “Prevalence and Diversity of Haemosporidian—Associated Matryoshka RNA Viruses in a Natural Population of Wild Birds”

**DOI:** 10.1002/ece3.73560

**Published:** 2026-05-14

Esperanza, C.W., Faircloth, C.E., Roy, S.W. and Sehgal, R.N.M. (2025), Prevalence and Diversity of Haemosporidian–Associated Matryoshka RNA Viruses in a Natural Population of Wild Birds. *Ecology and Evolution*, 15: e71239, https://doi.org/10.1002/ece3.71239

The following funding source was missing from the Funding statement: “S‐MIP‐23‐2 Grant of the Research Council of Lithuania awarded to Gediminas Valkiūnas.”

The Funding statement should read as follows:

**Funding:** This work was supported by NIH, SC3‐GM144187. NIH SFSU/UCSF Bridge to Doctorate Fellowship, T32‐GM142515. S‐MIP‐23‐2 Grant of the Research Council of Lithuania awarded to Gediminas Valkiūnas.

We apologize for this error.

