# Supplementary figures and images for: Prevalence and Diversity of Haemosporidian–Associated Matryoshka RNA Viruses in a Natural Population of Wild Birds
Source: Ecol Evol. 2025 May 26;15(5):e71239. doi: 10.1002/ece3.71239 (PMC12105915; doi:10.1002/ece3.71239)

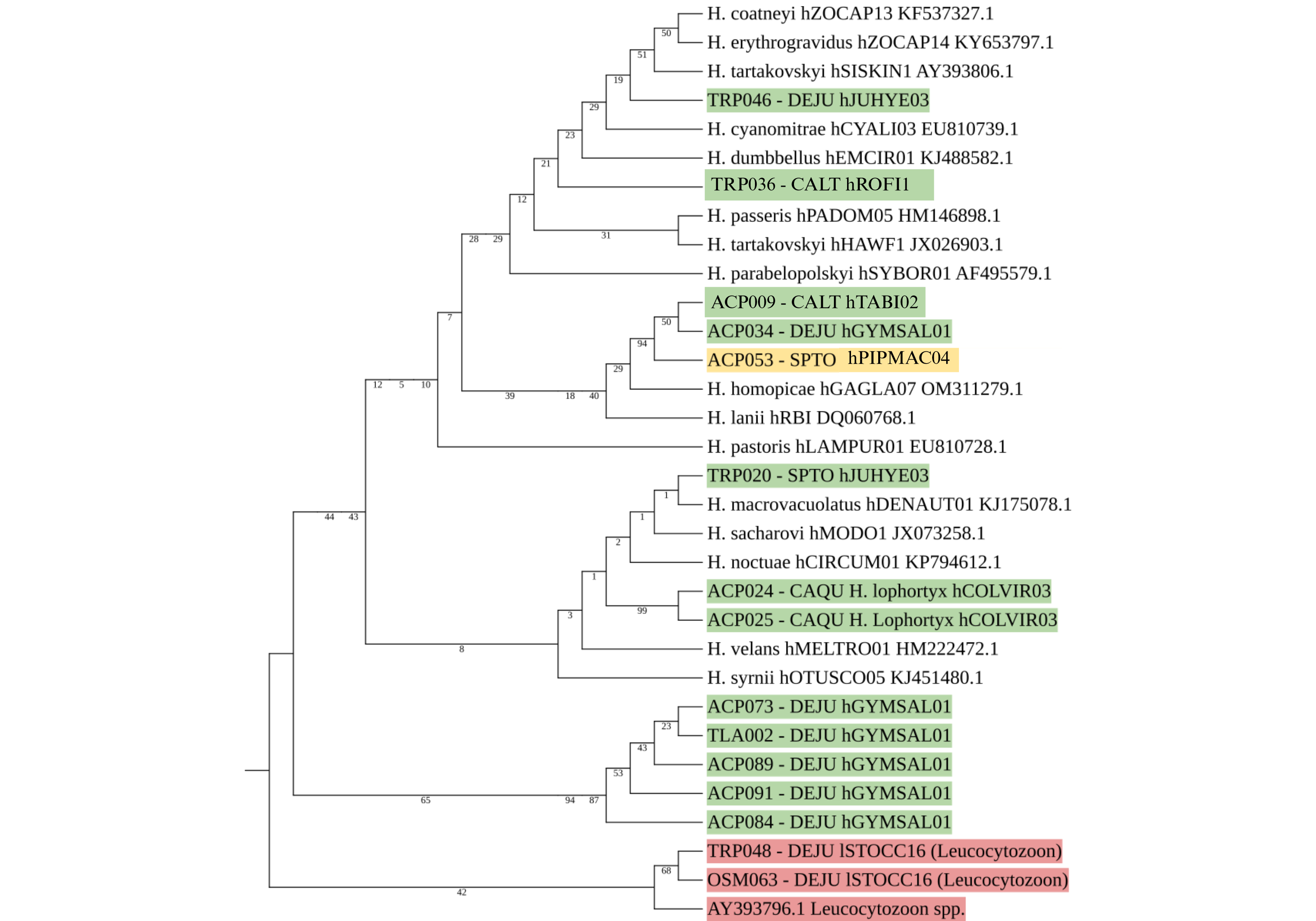

Supplement: Supplementary file 1 — Figure S1. [file ECE3-15-e71239-s002.png]

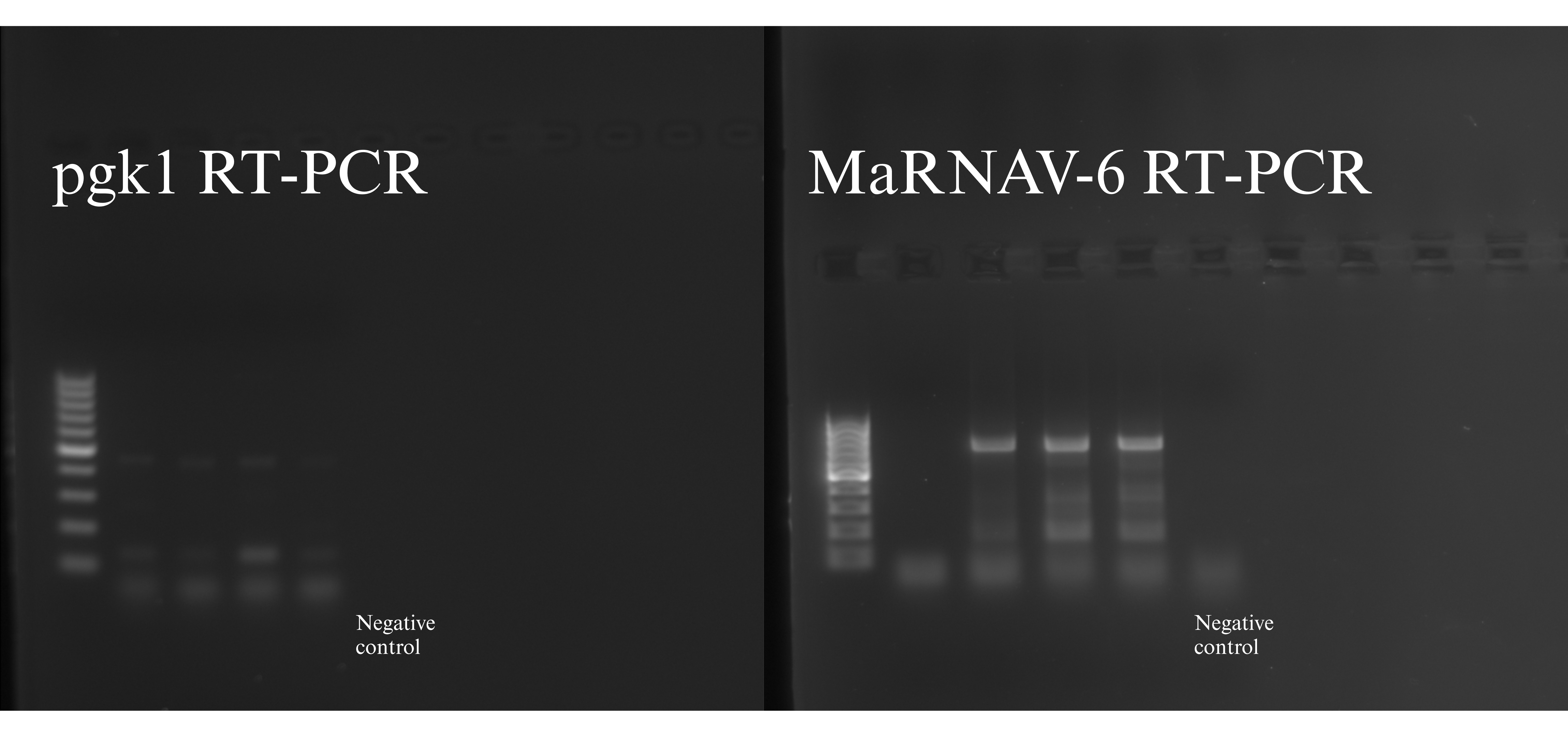

Supplement: Supplementary file 2 — Figure S2. [file ECE3-15-e71239-s001.png]

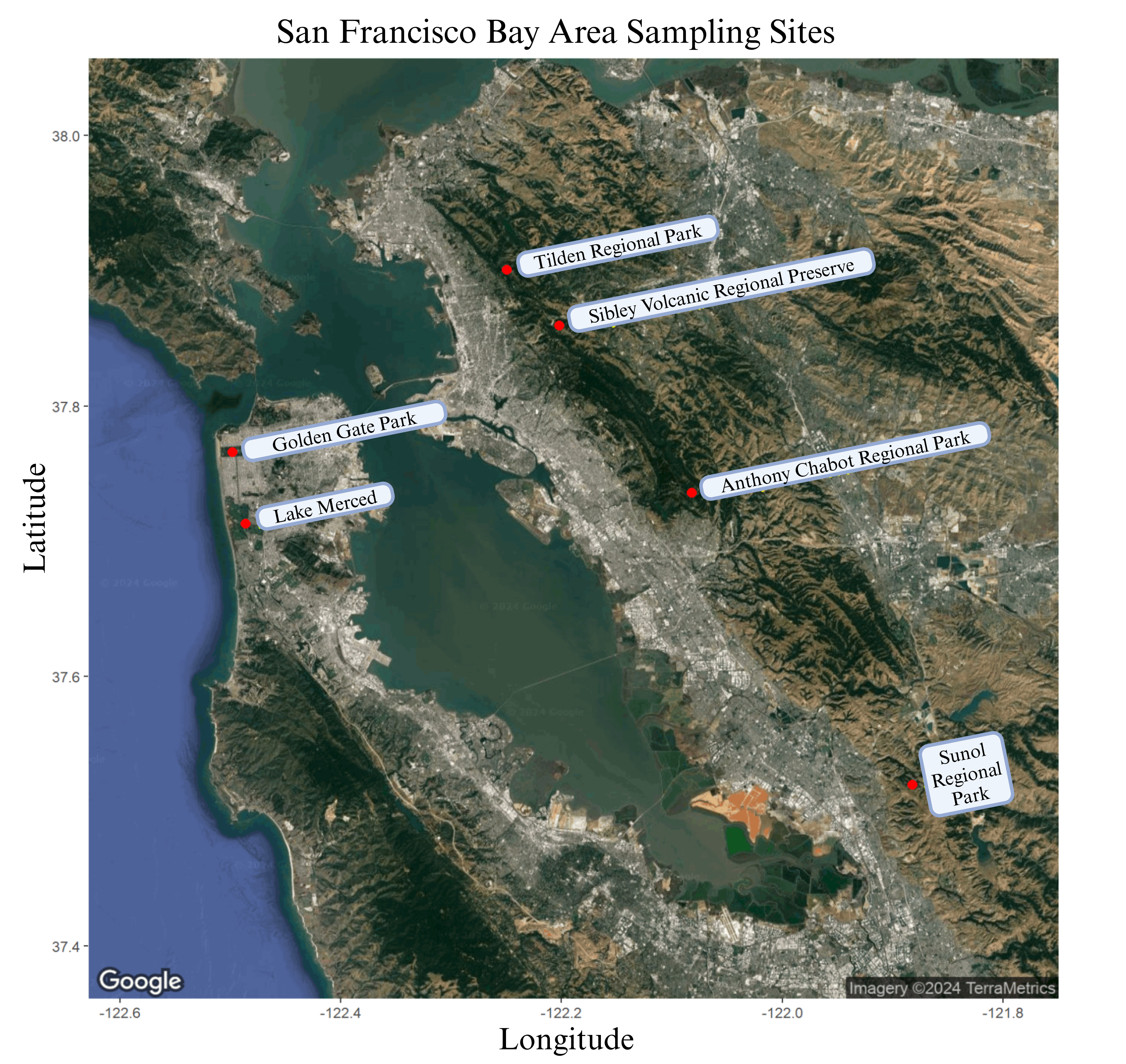

Supplement: Supplementary file 3 — Figure S3. [file ECE3-15-e71239-s006.png]
